# Digital mobility outcomes to describe real-world walking during recovery from a hip fracture: the Mobilise-D perspective

**DOI:** 10.1007/s41999-025-01391-w

**Published:** 2026-01-03

**Authors:** Clemens Becker, Tobias Eckert, Jochen Klenk, Carl-Philipp Jansen, Martin Aursand Berge, Monika Engdal, Beatrix Vereijken, Niki Brenner, Jorunn Helbostad, Ingvild Saltvedt, Lars Gunnar Johnsen, Hubert Blain, Valerie Driss, Lene Bergendal Solberg, Trine Strøm, Brian Caulfield, David Singleton, Judith Garcia-Aymerich, Laura Delgado-Ortiz, Sarah Koch, Joren Buekers, Paula Alvarez, Ram Miller, Daniel Rooks, Lynn Rochester, Silvia Del Din, Andrea Cereatti, Anna Marcuzzi

**Affiliations:** 1 Geriatric Center, Medical Faculty Heidelberg, Heidelberg University, Unit Digitale Geriatrie, UKHD, Heidelberg, Germany; 2 Robert Bosch Gesellschaft Für Medizinische Forschung mbH, Stuttgart, Germany; 3 Institute of Epidemiology and Medical Biometry, Ulm University, Ulm, Germany; 4 Department of Neuromedicine and Movement Science, Norwegian University of Science and Technology (NTNU), Trondheim, Norway; 5 Department of Geriatrics, St.Olavs Hospital HF, Trondheim, Norway; 6 Department of Orthopaedic Surgery, St.Olavs Hospital HF, Trondheim, Norway; 7 Centre d’Investigation Clinique, CHU Montpellier, Montpellier, France; 8 Department of Geriatrics, Montpellier University Hospital, MUSE, Montpellier, France; 9 Division of Orthopaedic Surgery, Oslo University Hospital, Oslo, Norway; 10 Insight Centre for Data Analytics, University College Dublin, Dublin, Ireland; 11 Barcelona Institute for Global Health (ISGlobal), Barcelona, Spain; 12 Universitat Pompeu Fabra (UPF), Barcelona, Spain; 13 CIBER Epidemiologia y Salud Publica (CIBERESP), Barcelona, Spain; 14 Department of Sport, Exercise and Health, University of Basel, Basel, Switzerland; 15 Translational Medicine, Biomedical Research, Novartis, Cambridge, MA USA; 16 Novartis Pharmaceuticals, Cambridge, MA USA; 17 Translational and Clinical Research Institute, Faculty of Medical Sciences, Newcastle University, Newcastle Upon Tyne, UK; 18 National Institute for Health and Care Research (NIHR) Newcastle Biomedical Research Centre (BRC), Newcastle University, Newcastle Upon Tyne, UK; 19 The Newcastle Upon Tyne Hospitals NHS Foundation Trust, Newcastle Upon Tyne, UK; 20 Department of Electronics and Telecommunications, Politecnico di Torino, Turin, Italy; 21 Department of Public Health and Nursing, Norwegian University of Science and Technology (NTNU), Trondheim, Norway; 22 Clinic of Rehabilitation, St. Olavs Hospital, Trondheim, Norway; 23 Inserm, CIC 1411, Montpellier, France

**Keywords:** Hip fracture, Digital mobility outcome, Wearable device, Walking

## Abstract

**Purpose:**

The burden of disease caused by fragility fractures, and hip fractures in particular, is significant both from a personal and a societal perspective. Digital technologies such as wearable devices now allow a real-world assessment of walking and physical mobility, describing temporal and spatial measures. Built on a transparent technical validation, the Mobilise-D consortium validated a set of digital mobility outcomes (DMOs) alongside a longitudinal clinical validation study (CVS). This article provides a description of DMOs along with supervised mobility assessment and patient-reported outcomes collected at entry of the CVS study in a sample of home-dwelling participants with a proximal femoral fracture (PFF) recruited within 1 year after surgery.

**Methods:**

Participants who underwent surgical treatment for a low-energy PFF were recruited from five sites in three high-income European countries. The first visit encompassed a clinical assessment (~ 3 h) and subsequent 7-day measurement of real-world mobility with a single wearable device. We categorized participants into four recovery phases according to the assessment time after surgery: acute phase (≤ 14 days after surgery, hospital stay), post-acute phase (15–42 days after surgery, mostly rehabilitation), extended recovery phase (43–182 days post-surgery, typically post-discharge at home), and long-term recovery phase (183–365 days post-surgery). All variables were reported descriptively for each phase using mean and standard deviation or median and 25th-75th percentile depending on their distribution.

**Results:**

We enrolled 564 PFF participants (66% women). The mean age was 77.5 (SD 9.6) years. Almost 90% of all participants (*n* = 505) had a valid digital mobility assessment (wear time of > 12 h per day and at least 3 valid days). DMO domains such as amount (e.g., number of steps), pace (e.g., walking speed), and pattern (bout distribution) demonstrated large differences across phases.

**Conclusions:**

The observed variation in walking amount and pace and pattern across recovery phases indicate that DMOs provide a granular analysis of real-world mobility of hip fracture survivors. When confirmed by longitudinal analyses, including results on minimal important differences, the use of selected DMOs will provide a novel approach for monitoring, predictive modelling, prognosis, stratification, and evaluation of clinical trials and hip fracture services.

## Key summary points

**Aim:**

To describe digital mobility outcomes in a sample of home-dwelling participants with a hip fracture at different phases of recovery (within 1 year from surgery)

**Findings:**

Overall, 90% of the 564 participants recruited had valid digital mobility assessment, indicating that this approach is highly feasible in this group. The amount (e.g., number of steps), pace (e.g., walking speed), and pattern (bout distribution) domains of the digital mobility assessment showed large differences across phases.

**Message:**

The observed variation in walking amount and pace and pattern across recovery phases indicate that digital mobility outcomes can provide an in-depth analysis of real-world mobility of hip fracture survivors.

## Background

The burden of disease caused by fragility fractures is increasing in most high-income countries [1, 2]. Of these, hip fractures (syn. proximal femoral fractures—PFF) are the most common and often the most severe injury type, with an estimated 30-day mortality rate of up to 10% [3]. Along with pre-existing comorbidities, hip fractures are also common causes for care home admissions affecting around 10% of all hip fracture survivors [4]. However, that the majority of them will return to their homes and have a life expectancy of several years is often overlooked [5, 6]. Thus, regaining sufficient mobility, self-care capacity, and getting adequate pain control can make a pivotal difference to survival, post-surgery care needs, and quality of life. However, frequent problems such as fear of falling, depression, pain, and fatigue need to be recognized and addressed [7]. By many experts, the frequent loss of independence following a hip fracture is considered inevitable rather than a consequence of insufficient rehabilitation content, duration, frequency, and intensity. Even high-income countries have widely disparate post-fracture services that range from almost none to moderate intensity rehabilitation. Thus, there is a need to focus on post-operative mobility to optimize disability-free survival perspectives [8, 9].

The most recent Cochrane Review evaluating the effect of rehabilitation programs for patients with PFF concluded that robust data on mortality and care home admissions are available. However, valid data on physical mobility and other core patient outcomes are missing or incomplete [10]. Emerging digital technologies such as inertial wearable devices enable granular measurement of mobility in patients with PFF in their real-world environment [11]. Recent developments in movement sensor algorithms led by the Mobilise-D consortium [12] allow the measurement of temporo-spatial parameters, such as walking speed, cadence, and stride length in walking bouts of different durations. In total, 24 digital mobility outcomes (DMOs) were technically validated, encompassing five domains of walking: amount, pattern, pace, rhythm, and bout-to-bout variability [13]. This expands the available data assessed in real-world environments and opens a new arena for evaluating the outcomes of surgical treatment, orthogeriatric co-management, and rehabilitation strategies such as home vs. inpatient rehabilitation. Further options include patient monitoring, stratification and predictive modeling [14].

This paper presents cross-sectional results of real-world DMOs and supervised clinical outcomes assessment (COA) in a large sample of PFF participants recruited within a year from surgery as part of the Mobilise-D Clinical Validation study (CVS) [15]. Also, patient-reported outcomes and characteristics are described, and considerations about the feasibility of digital mobility assessment in this clinical population are discussed.

## Methods

### Study design and setting

The Mobilise-D CVS is a multicenter observational cohort study which included participants who either had a PFF, Parkinson’s disease, chronic obstructive pulmonary disease, or multiple sclerosis. The study protocol for the Mobilise-D CVS was registered within the ISRCTN registry in December 2020 (Reference number: 12051706) and published [15]. All eligible participants were invited for a first visit and follow-up visits every 6 months for up to 24 months. This paper reports on data of the first visit of the 564 PFF participants included (database version 6.2).

Recruitment of PFF participants took place between May 2021 and June 2023 across five clinical sites: the Fracture Liaison Service of Centre Hospitalier Universitaire de Montpellier (CHUM) in France; Robert Bosch Hospital, Stuttgart and Heidelberg University Medical Center in Germany; St. Olavs Hospital, Trondheim University Hospital and Oslo University Hospital in Norway.

### Participants

Inclusion criteria were: surgical treatment for a low-energy fracture of the proximal femur (International Classification of Diseases (ICD-10) codes S72.0, S72.1, S72.2) within the previous year, 45 years of age or older, community dwelling prior to the hip fracture, able to walk 4 m independently at the time of the first assessment, and ability to comply with study procedures including providing informed consent and being able to read and write in the first language of the respective country. Those who had major heart surgery, stroke, active treatment for malignancy, major psychiatric disorders including severe dementia or delirium, or continued substance abuse within 3 months prior to study entry were excluded.

**Fig. 1:**
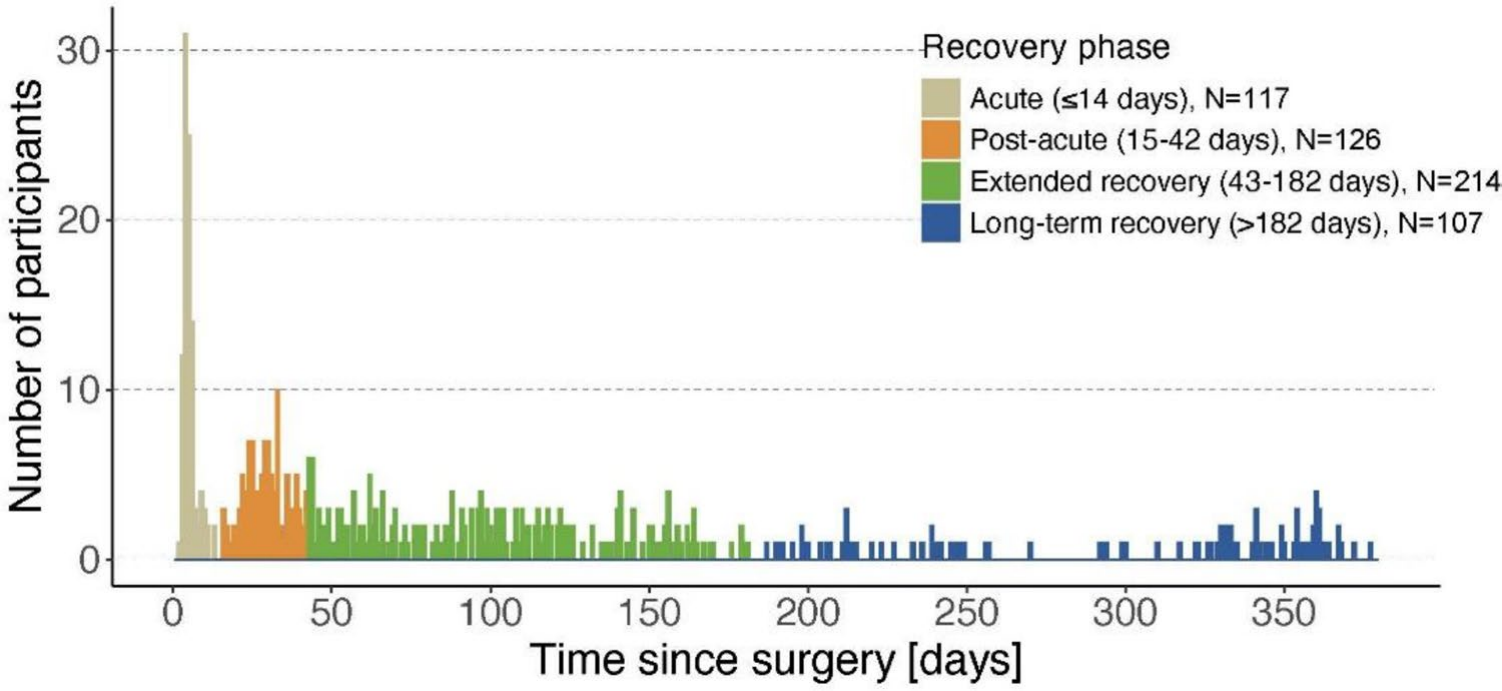
Inclusion timeline of the study sample into the study during the first post-operative year

Participants were allowed to enter the study at any time point during the first post-operative year, although efforts were made to include them as soon as possible after surgery. Based on setting (in- or outpatient) and evidence from an existing longitudinal observational study on functional recovery [16], participants were categorized into four phases according to the assessment time after surgery: the acute phase (≤ 14 days post-surgery, in-hospital stay), the post-acute recovery phase (15–42 days post-surgery, most often rehabilitation), the extended recovery phase (43–182 days post-surgery, typically post-discharge at home), and the long-term recovery phase (183–365 days post-surgery, representing the second half of the 1st year). The participants’ inclusion timeline according to these different phases is shown in Fig. 1.

### Study procedure

Participants were recruited during their hospital stay after surgery, at rehabilitation wards after hospital discharge, and through patient lists. After identification of potential participants, the first screening for eligibility was performed through medical note reviews. Those potentially eligible were approached either in person or by phone and provided with the patient information sheet and details about the study. Interested persons provided written informed consent and underwent a face-to-face screening appointment to determine eligibility. If included into the study, the first assessment took place within 2 weeks after the initial screening.

On-site assessment took around 3 h and could be split into two sessions if participants became fatigued. All data were entered directly to a tablet device that contained appropriate software applications designed to match the workflow of the protocol, and data were subsequently transferred to a centralised database for processing and analysis. At the end of the assessment, participants were provided with a single wearable device attached to the lower back. All assessors were trained prior to study start via a series of webinars and standardized materials. A detailed description of the study procedures and assessment battery was provided in the study protocol [15].

### Assessment battery

Participants’ characteristics collected included sociodemographic, i.e., age, sex, education, living situation and residence, pre-fracture functional status measured using the Nottingham Extended Activity of Daily Living Index (NEADL, range 0–66, higher scores indicate greater independence) [17], and pre-surgical health status measured by the American Society of Anaesthesiologists score (ASA, range I–V, higher scores indicate worse health status) [18]. Clinical characteristics included fracture type, days since surgery, and use of walking aids indoors and outdoors.

Supervised clinical outcome assessment was performed to assess physical capacity, including the Short Physical Performance Battery (SPPB, range 0–12, higher scores indicate better physical capacity) [19], comprising a static balance test, a 4-m walk test, and a five-repetition chair rise test (5CRT). Additionally, the timed-up-and-go test (TUG) [20] and the 6-min walk test (6MinWT) [21] were performed. The cognitive status at study entry was determined using the short Mini-Mental State Examination (sMMSE, range 0–6, higher scores indicate better cognitive status) [22].

Patient-reported outcomes included perceived function and disability measured by the Late-Life Function and Disability Instrument (LLFDI, range 0–100, higher scores indicate better functional level) [23], the Life-Space Assessment (LSA, range 0–120, higher scores indicate larger life space) [24], the Short Falls Efficacy Scale-International (Short FES-I, range 0–28, higher scores indicate greater concerns of falling) [25], the pain visual analog scale (VAS, range 0–100, higher scores indicate greater pain levels) [26], the Euro-QoL (EQ-5D) VAS score (range 0–100, higher scores indicate better health status) [27], and the Functional Assessment of Chronic Illness Therapy Fatigue Scale (FACIT, range 0–52, higher scores indicate less fatigue) [28].

Acute PFF participants required some protocol adaptations due to their post-surgical state. In this group, the LLFDI was administered to assess pre-fracture function instead of the ‘current status’, since this questionnaire has not been validated for acute patients. Also, acute participants did not carry out the 5CRT as part of the SPPB due to safety concerns. The 6 MinWT and the TUG were not feasible for most participants in the first post-operative days due to pain and physical limitations.

### Digital mobility outcome assessment

Digital assessment of real-world mobility was recorded over seven consecutive days using a single wearable device. The wearable device used was either the McRoberts MoveMonitor + (McRoberts B.V., The Hague, The Netherlands) or the Axivity AX6 (Axivity Ltd, Newcastle Upon Tyne, UK).

In total, 24 DMOs were technically validated in a prior study which included PFF participants, covering the domains of walking amount, pace, pattern, rhythm, and bout-to-bout-variability [29]. For this paper, we limit the reporting to the most commonly used DMOs, namely walking amount, pace, and pattern [30]. Parameters related to rhythm and bout-to-bout variability will be reported in an upcoming paper on construct validity of all DMOs.

Data from the single wearable device were first standardized according to the data structure outlined elsewhere [31]. Walking bouts (WB) were identified [32], and gait features such as initial foot contact, cadence, and stride length were extracted using the validated Mobilise-D computational pipeline [12, 33]. Six DMOs were calculated for each WB: duration, number of steps, cadence, walking speed, stride length, and stride duration. Days or weeks that did not meet the predefined minimum wear time, i.e., > 12 h required for a valid day (during waking time from 07:00 to 22:00 h) and ≥ 3 valid days for a reliable week (no weekday/weekend restrictions), were removed [34]. Bout level DMOs were aggregated at the weekly level: number of steps (n), walking duration (minutes), walking speed (m/s) in shorter (10-30 s) and longer (> 30 s) WBs, 90th percentile (P90) walking speed (m/s) in WBs of > 10 s and > 30 s, stride length in WBs of 10-30 s and > 30 s (cm), number of WBs (all, > 10 s, > 30 s, > 60 s), WB duration (s), and P90 WB duration (s) [29].

The technical validation also demonstrated that patients with low physical function and very slow walking speed < 0.5 m/s had a higher error rate for the spatial parameters such as stride length and thereby walking speed [13, 35]. It was anticipated that this bias would affect measurements in acute participants to a greater extent compared to participants in other phases due to a short stride length and thereby a lower walking speed.

### Statistics

For sociodemographic, clinical, patient-reported outcomes, and DMOs, time interval-based participant characteristics are presented as means and standard deviations (SD) or median and 25th and 75th percentiles (P25-P75) depending on their distribution. Categorical variables are presented as numbers and percentages. Boxplots were used to visualize the distribution of DMOs across the four recovery phases. The statistical software R (v4.4.0; R Core Team 2024) was used for the analyses.

## Results

### Main characteristics

Table 1 presents the demographic and pre-fracture status of the sample. In total, 564 participants (mean (SD) age: 77.5 (9.6) years) were included, of which the majority were females (66.3%). Overall, 63 participants were younger than 65 years (12.5%). Participants had relatively independent pre-fracture physical function with a median (P25-P75) NEADL score of 60.0 (51.0–64.0), reflecting a sample with little to moderate impairment. Also, more than half were classified with ASA scores of III and IV (52.3%) indicating major to severe systemic comorbidities (Table 1).

**Table 1:** Sociodemographic characteristics and pre-fracture status of the study sample

Domain	Variable	All	Acute	Post-acute	Extended recovery	Long-term recovery
Sociodemographic domain	Participants, *n*	564	117	126	214	107
	Age, years, mean (SD)	77.5 (9.6)	78.4 (9.2)	80.0 (9.3)	75.8 (9.6)	77.2 (9.6)
	Sex, females, *n* (%)	374 (66.3)	81 (69.2)	82 (65.1)	138 (64.5)	73 (68.2)
	Education, *n* (%)					
	College degree or higher	264 (46.8)	29 (24.8)	75 (59.5)	102 (47.7)	58 (54.2)
	High school	151 (26.8)	41 (35.0)	28 (22.2)	60 (28.0)	22 (20.6)
	Less than high school (≤ 10 yrs)	146 (25.9)	46 (39.3)	23 (18.3)	51 (23.8)	26 (24.3)
	Not defined	3 (0.5)	1 (0.9)	0 (0.0)	1 (0.5)	1 (0.9)
	Living situation, *n* (%)					
	Alone	270 (47.9)	60 (51.3)	66 (52.4)	87 (40.7)	57 (53.3)
	Spouse/partner	256 (45.4)	51 (44.4)	54 (42.9)	109 (50.9)	41 (38.3)
	Family and child	31 (5.5)	3 (2.6)	5 (4.0)	16 (7.5)	7 (6.5)
	Others	7 (1.3)	2 (1.7)	1(0.8)	2 (1.0)	2 (1.9)
	Residence, *n* (%)					
	Apartment	296 (52.5)	58 (49.6)	69 (54.8)	121 (56.5)	48 (44.9)
	House	248 (44.0)	57 (48.7)	52 (41.3)	85 (39.7)	54 (50.5)
	Independent living unit	16 (2.8)	2 (1.7)	4 (3.2)	7 (3.3)	3 (2.8)
	Others (including temporary care)	4 (0.8)	0 (0.0)	1 (0.8)	1 (0.5)	2 (1.9)
	Recruitment site, *n* (%)					
	Montpellier (France)	61 (10.8)	9 (7.7)	2 (1.6)	24 (11.2)	26 (24.3)
	Trondheim/Oslo (Norway)	261 (46.3)	103 (88.0)	32 (25.4)	87 (40.7)	39 (36.4)
	Stuttgart/Heidelberg (Germany)	242 (42.9)	5 (4.3)	92 (73.0)	103 (48.1)	42 (39.3)
Pre-fracture status	Pre-fracture NEADL total score [0–66], median (P25-P75)	60.0 (51.0–64.0)	60.0 (51.0–63.0)	58.5 (51.8–63.0)	59.5 (48.8–66.0)	62.0 (54.0–66.0)
	ASA score^a^, *n* (%)					
	I: healthy	48 (8.5)	6 (5.1)	7 (5.6)	21 (9.8)	14 (13.1)
	II: mild systematic disease	209 (37.1)	52 (44.4)	36 (28.6)	79 (36.9)	42 (39.3)
	III: severe systematic disease	259 (45.9)	48 (41.0)	72 (57.1)	96 (44.9)	43 (40.2)
	IV: incapacitating disease	36 (6.4)	10 (8.5)	10 (7.9)	12 (5.6)	4 (3.7)

SD: standard deviation; NEADL: Nottingham Extended Activity of Daily Living; ASA: American Society of Anesthesiologists; P25: 25th percentile; P75: 75th percentile. ^a^ *n* = 552 with complete data on ASA score

The adherence to the digital mobility outcome assessment was high, with a total of 505 participants (88.9%) providing valid data (i.e., at least 12 h during waking hours and ≥ 3 valid days wear time).

**Fig. 2:**
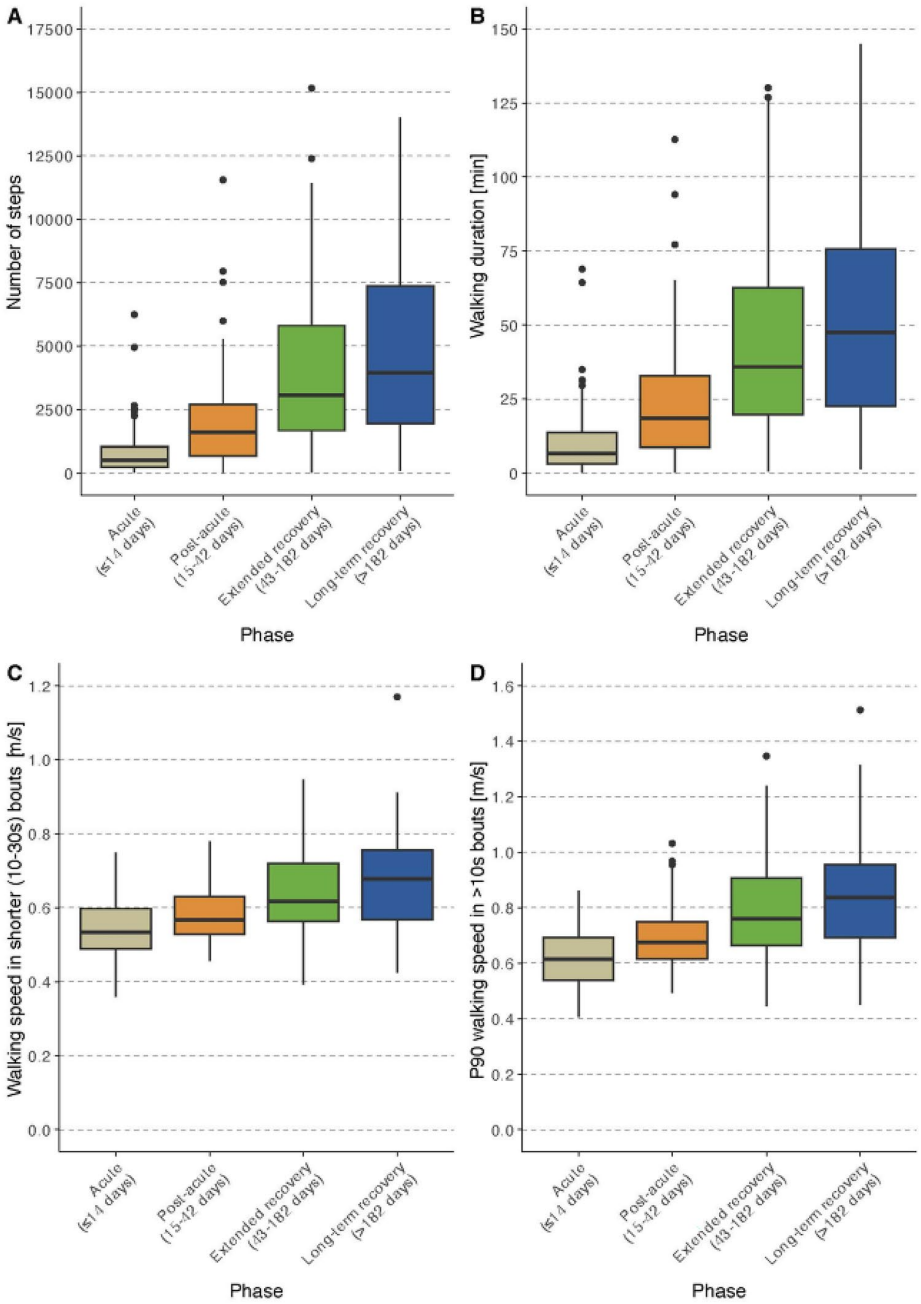

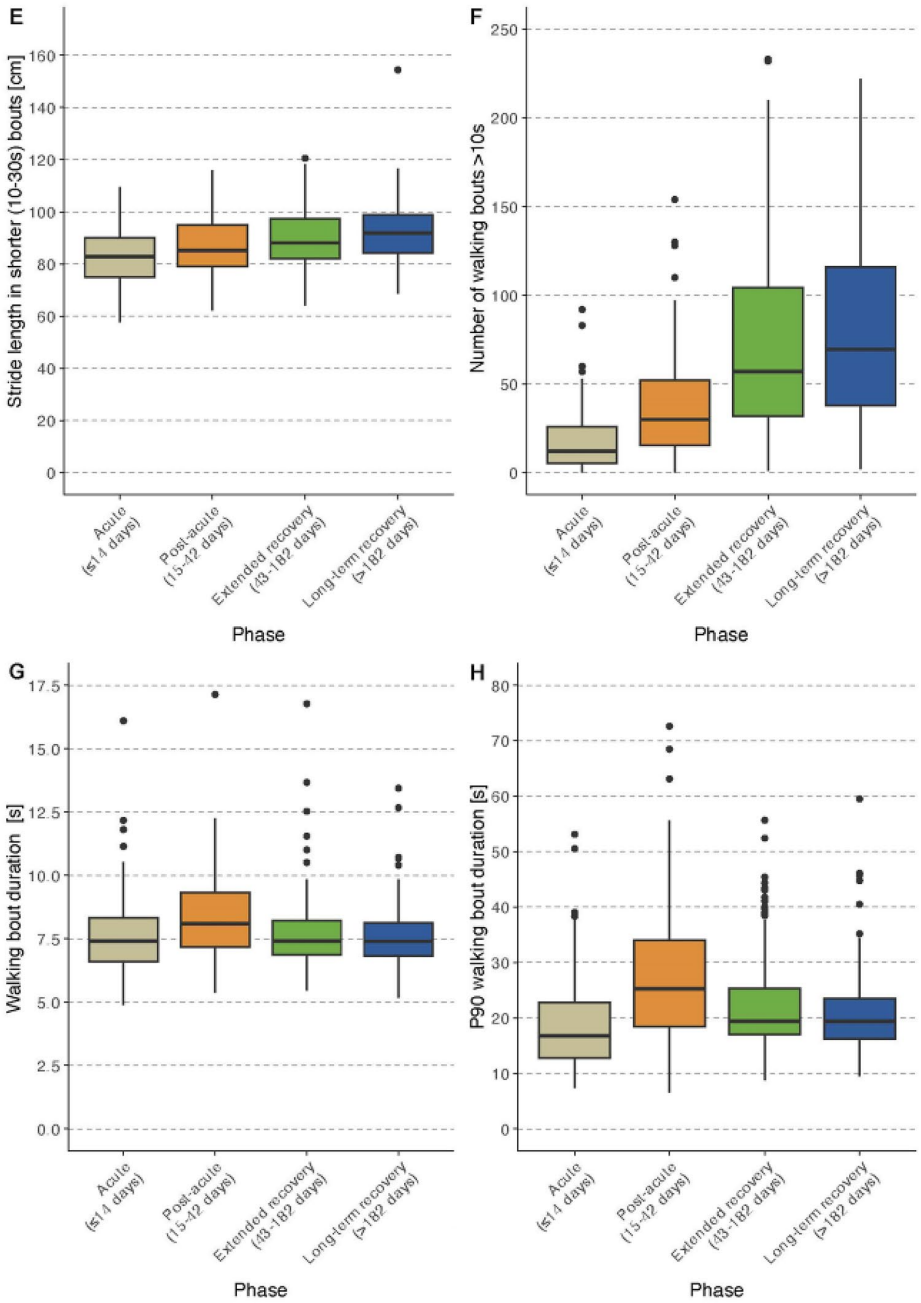
Boxplots for selected digital mobility outcomes stratified by recovery phases. Panels A-B report volume parameters. Panels C-E report pace parameters. Panels F–H report pattern parameters

Results for patient-reported outcomes, clinical outcome assessment, and DMOs are described for each recovery phase separately below and are reported in Tables 2 and 3. Selected DMOs stratified by the four recovery phases are shown in boxplot in Fig. 2.

**Table 2:** Clinical status, patient-reported outcomes, and supervised outcomes of the study sample stratified by phases

Domain	Variable	All *N* = 564	Acute *N* = 117	Post-acute *N* = 126	Extended recovery *N* = 214	Long-term recovery *N* = 107
Clinical Domain	Fracture type, *n* (%)					
	Femoral neck fracture	388 (68.8)	98 (83.8)	76 (60.3)	139 (65.0)	75 (70.1)
	Trochanteric fracture	176 (31.2)	19 (16.2)	50 (39.7)	75 (35.2)	32 (29.9)
	Days since surgery, median (P25-P75)	61.3 (21.8–144.3)	4.6 (3.4–5.4)	28.4 (23.4–35.4)	96.9 (64.9–129.1)	298.3 (211.4–348.4)
	Use of walking aids, n (%)					
	Indoors	269 (59.9)*	n.a**	114 (90.5)	120 (56.1)	35 (32.7)
	Outdoors	328 (73.1)*	n.a**	118 (93.7)	152 (71.0)	58 (54.2)
Patient-reported outcomes	LLFDI function, mean (SD)	51.8 (12.6)*	n.a**	45.4 (10.4)	52.8 (12.4)	57.4 (12.3)
	LSA [0–120], median (P25–P75)	38 (18.0-64.0)	n.a***	18.0 (8.0– 30.0)	43.5 (24.4– 64.1)	59.0 (32.5–81.5)
	sFES-I [7–28], mean (SD)	11.1 (4.5)	11.6 (5.2)	12.0 (4.4)	10.8 (4.3)	10.1 (4.1)
	Pain while walking [0–100], median (P25-P75)	25.0 (7.0–50.0)	50.0 (28.5– 70.0)	22.5 (8.0–50.0)	20.0 (3.0–44.8)	14.5 (1.0– 35.3)
	EQ-5D VAS [0–100], mean (SD)	62.5 (20.3)	52.2 (21.1)	60.5 (17.0)	65.1 (18.9)	70.6 (20.8)
	FACIT-F [0–52], mean (SD)	37.3 (10.4)	34.8 (10.0)	35.2 (11.2)	38.8 (10.0)	39.6 (9.9)
Physical capacity and cognitive screening	SPPB total score [0-12], mean (SD)	6.1 (3.2)	3.3 (2.0)	5.3 (2.6)	7.3 (2.9)	7.8 (3.3)
	4-meter gait speed, m/s, mean (SD)	0.68 (0.39)	0.35 (0.20)	0.61 (0.23)	0.79 (0.35)	0.91 (0.40)
	5CRT, s, mean (SD)	16.7 (6.5)	n.a***	17.3 (6.5)^a^	17.4 (7.1)^b^	15.1 (4.9)^c^
	TUG, s, mean (SD)	20.0 (12.8)	n.a***	26.2 (16.1)^d^	18.1 (10.3)^e^	17.0 (10.8)^f^
	6MinWT, meters, mean (SD)	284 (129)	n.a***	221 (105)^g^	299 (127)^h^	320 (131)^i^
	sMMSE (0–6), mean (SD)	5.2 (1.1)	5.2 (1.1)	5.0 (1.1)	5.3 (1.0)	5.2 (1.1)

Number of completed tests in 5CRT, TUG, 6MinWT

^a^ *n* = 28

^b^ *n* = 143

^c^ *n* = 103

^d^ *n* = 116

^e^ *n* = 208

^f^ *n* = 106

^g^ *n* = 112

^h^ *n* = 208

^i^ *n* = 106.

*n = 449, exclusion of acute participants; **n.a. = not applicable, only pre-fracture status available; ***n.a. = not applicable, not performed in acute participants

P25: 35th percentile; P75: 75th percentile; SD: standard deviation; LLFDI: late-life function disability instrument; LSA: life-space assessment; sFES-I: Short Falls Efficacy Scale-International; EQ-5D VAS: EuroQoL visual analog scale; FACIT-F: Functional Assessment of Chronic Illness Therapy-Fatigue Scale; SPPB: Short Physical Performance Battery; CRT: chair rise test; TUG: time up and go; 6MinWT: 6-min walk test; sMMSE: Mini-Mental State Examination

**Table 3:** Digital mobility outcomes of the study sample

Variables	All *N* = 505	Acute *N* = 99	Post-acute *N* = 116	Extended recovery *N* = 202	Long-term recovery *N* = 88
Amount					
Number of steps	2139 (824–4603)	515 (233–1038)	1595 (681–2704)	3063 (1669–5805)	3949 (1937–7360)
Walking duration, min	25.2 (10.2–50.4)	6.6 (3.0–13.8)	18.6 (8.4–32.4)	36.0 (19.8–62.4)	47.4 (22.8–75.6)
Pace					
Walking speed in shorter (10–30 s) WBs, m/s	0.60 (0.54–0.69)	0.53 (0.49–0.60)	0.57 (0.53–0.63)	0.62 (0.56–0.72)	0.68 (0.42–0.57)
Walking speed in longer (> 30 s) WBs, m/s	0.65 (0.58–0.80)	0.56 (0.51–0.60)	0.62 (0.56–0.70)	0.70 (0.60–0.84)	0.80 (0.66–0.88)
P90 walking speed in WBs > 10 s, m/s	0.71 (0.62–0.85)	0.62 (0.54–0.69)	0.68 (0.62–0.75)	0.76 (0.66–0.91)	0.84 (0.69–0.96)
P90 walking speed in longer (> 30 s) WBs, m/s	0.71 (0.63–0.89)	0.59 (0.55–0.66)	0.68 (0.61–0.78)	0.78 (0.66–0.96)	0.89 (0.71–1.02)
Stride length in shorter (10–30 s) WBs, cm	87.4 (80.4–95.7)	82.9 (75.0–89.9)	85.2 (79.0–95.1)	88.1 (82.0–97.4)	91.8 (84.2–98.9)
Stride length in longer (> 30 s) WBs, cm	95.5 (85.7–107.6)	84.6 (78.4–93.3)	90.9 (82.7–105.9)	97.9 (89.6–109.3)	103.4 (95.2–111.5)
Pattern					
Number of WBs	125 (50–236)	35 (21–76)	83 (37–143)	190 (113–294)	231 (119–372)
Number of WBs > 10 s	43 (18–823)	12 (6–26)	30 (16–52)	57 (32–104)	70 (38–116)
Number of WBs > 30 s	7 (2–14)	1 (0–4)	7 (3–12)	10 (4–18)	10 (4–18)
Number of WBs > 60 s	2 (0–5)	0 (0–1)	2 (0–4)	3 (1–6)	4 (1–7)
WB duration, s	7.5 (6.9–8.4)	7.4 (6.6–8.3)	8.1 (7.2–9.3)	7.4 (6.9–8.2)	7.4 (6.8–8.1)
P90 WB duration, s	19.7 (16.2–26.5)	16.8 (12.7–22.8)	25.2 (18.4–34.0)	19.4 (17.0–25.3)	19.4 (16.3–23.6)

All values are reported as median with 25th and 75th percentiles

WB: walking bout; P90: 90th percentile

### Acute phase participants (≤ 14 days post-operatively)

Acute participants (*n* = 117, mean (SD) age 78.4 (9.2) years, 69% women) were mostly recruited in Trondheim. Eighty-four percent had a femoral neck fracture (Table 2).

### Patient-reported outcomes and clinical outcome assessment

The mean (SD) s-FESI was 11.6 (5.2) indicating moderate concerns about falling. Participants reported severe pain while walking (median (P25–P75) VAS: 50.0 (28.5–70.0)) and rated their overall health on the EQ-5D VAS as 52.2 (21.1), reflecting a moderately impaired quality of life. The mean (SD) FACIT score was 34.8 (10.0) indicating moderate levels of fatigue. Cognitive status was robust with a mean (SD) sMMSE of 5.2 (1.1), which translates to an MMSE score in the range of 26 points or above [36]. The post-operative assessments of mobility showed a very low physical capacity in this group (mean (SD) supervised 4-m gait speed: 0.35 (0.20) m/s) and mean (SD) for the SPPB of 3.3 (2.0) (Table 2).

### DMOs

The median (P25-P75) step count was 515 (233–1,038) steps/day and walking time was 6.6 (3.0–13.8) min. For short WBs (10–30 s), the median (P25-P75) walking speed was 0.53 (0.49–0.60) m/s and P90 walking speed was 0.62 (0.54–0.69) m/s. The median (P25-P75) walking speed for walking bouts of > 30 s was 0.56 (0.51–0.60) m/s. For bout distribution, a median (P25-P75) number of 35 WBs (21–76) was identified with a median (P25-P75) duration of 7.4 (6.6–8.3) s and P90 duration of 16.8 (12.7–22.8) s. Further details are given in Table 3.

### Post-acute phase participants (15–42 days post-operatively)

Post-acute participants (*n* = 126, mean (SD) age 80.0 (9.3) years, 65% women) were mostly recruited in Stuttgart. In this group, nearly two-thirds had a femoral neck fracture (60.3%). More than 90% used walking aids indoors and outdoors (Table 2).

### Patient-reported outcomes and clinical outcome assessment

The mean (SD) s-FESI was 12.0 (4.4), indicating persistent moderate concerns about falling. Participants reported mild pain while walking (median (P25–P75) VAS: 22.5 (8.0–50.0)) and rated their overall health on average as 60.5 years (17.0). A mean (SD) LLFDI functional score of 45.4 (10.4) and a median (P25-P75) LSA score of 18.0 (8.0–30.0) indicated a low functional level and restricted life space in this group. The cognitive status and fatigue score were similar to the acute phase (mean (SD) sMMSE: 5.0 (1.1) and mean (SD) FACIT: 35.2 (11.2), respectively).

The post-acute assessments of mobility capacity showed a mean (SD) SPPB total score of 5.3 (2.6), indicating a low physical capacity, and a mean (SD) supervised 4-m gait speed of 0.61 (0.23) m/s. The mean (SD) distance walked in the 6MinWT was 221 (105) m (completers *n* = 112, 88.9%), and the mean (SD) time for completion of the 5 CRT was 17.3 (6.5) s (completers = 28, 22.2%); the TUG took on average 26.2 (16.1) s (completers = 116; 92.1%) (Table 2).

### DMOs

Participants recruited in the post-acute phase had a median (P25–P75) step count of 1595 (681–2704) steps/day and median (P25-P75) walking duration of 18.6 (8.4–32.4) min. For shorter WBs (10–30 s), the median (P25-P75) walking speed was 0.57 (0.53–0.63) m/s and the P90 walking speed was 0.68 (0.62–0.75) m/s. Median (P25–P75) walking speed was somewhat higher during longer walking bouts (> 30 s) compared to short WBs with 0.62 (0.56–0.70) m/s. The bout distribution identified a median (P25–P75) number of 83 WBs (37–143) with a median (P25-P75) duration of 8.1 (7.2–9.3) s and P90 duration of 25.2 (18.4–34.0) (Table 3).

### Extended recovery phase participants (43–182 days post-operatively)

Participants in this phase (*n* = 214, mean (SD) age 75.8 (9.6) years, 65% women) were recruited across all sites. Nearly two-thirds (65%) had a femoral neck fracture. Most participants used walking aids indoors (56.1%) and outdoors (71.0%) (Table 2).

### Patient-reported outcomes and clinical outcome assessment

The mean (SD) s-FESI was 10.8 (4.3), indicating moderate concerns about falling. Participants reported mild pain while walking (median (P25–P75) VAS: 20.0 (3.0–44.8)) and rated their overall health on the EQ-5D VAS as 65.1 (18.9). The mean (SD) LLFDI functional score of 52.8 (12.4) indicated a reduced functional level and the median (P25–P75) LSA score of 43.5 (24.4 to 64.1) reflects a mostly homebound lif-space in this group. The mean (SD) FACIT score was 38.8 (10.0), indicating moderate levels of fatigue. The cognitive status for this group was similar to the previous groups with a mean (SD) sMMSE: 5.3 (1.0).

The mean (SD) SPPB total score was 7.3 (2.9), indicating moderate functional limitations, and the mean (SD) supervised 4-m gait speed was 0.79 (0.35) m/s. The mean (SD) distance walked in the 6MinWT was 299 (127) m (completers = 199; 91.7%), the mean (SD) time for 5 CRT was 17.4 (7.1) s (completers *n* = 143; 65.9%), and the TUG took on average 18.1 (10.3) s (completers = 208; 95.9%) (Table 2).

### DMOs

This group had a median (P25–P75) step count of 3063 (1669–5805) steps/day and median (P25–P75) walking time of 36.0 (19.8–62.4) min. For short WBs (10–30 s), the median (P25–P75) walking speed was 0.62 (0.56–0.72) m/s and the P90 walking speed was 0.76 (0.66–0.91) m/s. The median (P25–P75) walking speed was higher during longer walking bouts (> 30 s) with 0.70 (0.60–0.84) m/s. For bout distribution, a median (P25–P75) number of 190 WBs (113–294) were identified with a median (P25–P75) duration of 7.4 (6.9–8.2) s and P90 duration of 19.4 (17.0–25.3) s (Table 3).

### Long-term recovery phase participants (183–365 days post-operatively)

Participants in this group were recruited across all sites (*n* = 107, mean (SD) age 77.2 (9.6) years, 68% women). More than two-thirds (70.1%) had a femoral neck fracture. Fewer participants in this group used walking aids indoors (32.7%), but more than half used them outdoors (54.2%) (Table 2).

### Patient-reported outcomes and clinical outcome assessment

The mean (SD) s-FESI was 10.1 (4.1), indicating moderate concerns about falling. Participants reported mild pain while walking (median (P25–P75) VAS: 14.5 (1.0–35.3)). The overall health was rated on average as 70.6 (20.8), showing the highest value of the four groups. The mean (SD) LLFDI functional score of 57.4 (12.3) indicates a higher functional level than the extended recovery group. The median (P25–P75) LSA score of 59.0 (32.5–81.5) indicates a life space including more outdoor activities in this group. The mean (SD) FACIT score of 39.6 (9.9) still reflects moderate fatigue level. The cognitive status was similar to the other groups (mean (SD) sMMSE: 5.2 (1.1)).

The assessments of mobility capacity showed a mean (SD) SPPB score of 7.8 (3.3), indicating moderate functional limitation, and a mean (SD) supervised 4-m gait speed of 0.91 (0.40) m/s. The mean (SD) distance walked in the 6MinWT was 320 (131) m (completers = 103; 96.2%), the mean (SD) time for the 5 CRT was 15.1 (4.9) s (completers = 74; 69.2%), and the TUG took on average 17.0 (10.8) s (completers = 106; 99.1%) (Table 2).

### DMOs

Participants recruited in the long-term recovery phase had a median (P25–P75) step count of 3949 (1937–7360) steps/day and a median (P25–P75) walking time of 47.4 (22.8–75.6) min. For short WBs (10–30 s), the median (P25–P75) walking speed was 0.68 (0.42–0.57) m/s, and the P90 walking speed was 0.84 (0.69–0.96) m/s. The median (P25-P75) walking speed was higher during longer WBs (> 30 s) compared to short WBs with 0.80 (0.66–0.88) m/s. For bout distribution, a median (P25-P75) number of 231 WBs (119–372) were identified with a median (P25-P75) duration of 7.4 (6.8–8.1) s and P90 duration of 19.4 (16.3–23.6) s (Table 3).

## Discussion

### Main findings and lessons learned

We successfully recruited more than 500 patients with PFF within a year from surgery and assessed their functional capacity and real-world mobility performance across four different, time-based recovery phases. Overall, we found that assessment of real-world mobility was highly feasible in this cohort, with nearly 90% of participants completing the 7-day digital mobility assessments. We also found that commonly used mobility assessments had limited feasibility during the acute and post-acute phases due to floor effects. This included the 5CRT, the TUG test and to a smaller degree the 6MinWT. Vice versa, some of these measures such as the 4-m walk test or a 6MinWT could often not be performed in home environments which limits their usefulness in future trials requiring home visits or remote study designs.

As expected, we observed major differences in the amount, pace, and pattern of walking using wearable devices between participants in the four phases. Walking amount such as the number of steps and walking time, walking speed, and number of WBs were much higher in participants recruited at later post-operative phases compared to those in earlier phases. These differences became smaller but were still noticeable for those recruited in the second half of the year after surgery compared to the participants during the first 6 months. However, these differences were not found for all DMOs. The average walking bout duration was longer during the post-acute phase compared to the bout duration after discharge probably due to longer corridors during the inpatient rehabilitation phase. Therefore, some of the differences are likely to be caused by factors such as the built environment, neighborhood walkability, traffic, green space, and environmental factors such as rain, heat, icy conditions, or air pollution.

Compared to a community-dwelling cohort of older adults in the InChianti study (Albites-Sanabria 2025 under review), the DMOs data demonstrate that there still is considerable gap in the amount and pattern of walking between even the best quartile of patients with PFF and healthy controls. The InChianti cohort with a mean age of 79 years had a median value of 8261 steps (vs 3949), and a walking time of 90 min (vs. 48 min). Around 155 walking bouts of 10 s-30 s (vs 70) and 26 walking bouts with a duration of 30 s and longer were detected (vs 10). The gait speed parameters did not differ. The walking speed was 0.64 m/s in shorter (vs. 0.68) and 0.79 m/s in longer walking bouts (vs. 0.8).

### Take home messages for clinical trialists and regulators

Due to the pan-European decrease in length of stay post-operatively, it will be increasingly challenging to enroll patients with PFF in the acute phase particularly for randomized controlled studies. Most patients were requesting to talk to their family before entering the study. Many potential participants explained that they wanted to get back to their home environment before entering the study. Our data also show that the group recruited during the post-acute phase was somewhat older and had more comorbidities compared to the acute phase. This must be considered to maximize external validity.

The ongoing preferred use of walking amount parameters (step counts and walking duration) is based on outdated technology and ignores the lack of spatiotemporal information (stride length and walking speed) which is relevant from a regulatory, patient and clinical perspective. We therefore recommend reporting spatial *and* temporal information to describe cross-sectional and longitudinal trajectories of patients. We also recommend the use of mean and maximum pace information in longer (30 s+) and shorter walking bouts (10–30 s). Longer walking bouts represent mostly outdoor activities. Shorter walking bouts are more informative on indoor walking. Additional analyses and methods such as EMA (ecological momentary assessments) are needed for this ground truth-based validation. Maximum pace informs about capacity and the potential to adapt walking speed when needed, for example when navigating pedestrian crossings. The pattern recognition (bout frequency in shorter and longer bouts) gives insight into the functional recovery with an anticipated increase in the number of walking bouts with longer duration. The description of maximum walking bout duration informs about habit formation and capacity.

In a recent systematic review on all European Medicines Agency decisions that included bone health medication [37], we could not identify any study that included supervised *or* digital mobility assessments as part of the regulatory process. Physical mobility is impaired in most osteoporotic populations, with PFF being the most pronounced clinical example. The final acceptance of wearable technologies will need data from randomized controlled trials [11]. In our discussions with health technology assessment bodies, it has been questioned whether an improvement in physical mobility is a healthcare claim at this moment. Our data of the PFF cohort point in the opposite direction. It is undisputed that an increase in walking amount has numerous beneficial health effects [38], making a strong case for its mandated assessment. The analysis of pace is needed since clinically measured gait speed was found to be a strong predictor of long-term rehabilitation success [39]. An even higher predictive validity for clinical events is assumed for measures under real life conditions.

### Take home messages for clinical experts and patients

The challenge of using DMOs (and COAs) in clinical practice and the derived empowerment of patients lie in the interpretation of single results (*n* = 1) and comparing the results with known groups of similar patients. The recovery phases presented in this study were chosen to give reasonable feedback to different professional groups that are in charge and involved with patients in these different time frames, such as orthopedic surgeons, geriatricians, physiotherapists, occupational therapists, nursing staff, and general physicians. The (repeated) digital assessments can describe the performance based on walking amount, pace and pattern. This can provide guidance on whether reassurance, watchful waiting, or action such as training boosters is appropriate. The boxplot diagrams and the quartiles listed give an indication on how patients with PFF regain mobility post-operatively. When a repeated assessment is performed, it can inform about a change in the expected trajectory, including improvement and worsening of the physical performance [11].

From a patient and/or family caregiver perspective, it is clear that physical mobility is a core part of quality of life and determines the probability of an independent lifestyle and the fundamental question of the possibility to return to the pre-fracture state. The core domains of patient perspectives on mobility include ease and effort of walking, the perceived safety, pace, and walking distance [40]. The presented data provide insight into how this compares to other patients with PFF. Future longitudinal data analyses will also allow the identification of patients who do not follow expected trajectories. The DMOs can describe pace, pattern, and amount, whereas the perception of safety and effort of walking requires the administration of patient-reported outcome measures. This process will require further co-design work with patients and health care professionals [41].

## Limitations

The pre-pandemic ambition was to recruit an acute PFF population and follow as many as possible over a period of 24 months. When the COVID-19 pandemic hit, recruitment and assessment had to be postponed or adapted in order to adhere to the medical circumstances and comply with the legal obligations which resulted in a longer recruitment period. Furthermore, our study did not include care home residents and patients with moderate to severe dementia and major delirium. The inclusion of younger patients (i.e. < 65 years, 12% of the sample) was accepted to harmonize the study protocol with the other disease cohort under the condition that they had a low energy trauma. All these decisions were defined a priori and in accordance with the study protocol. Consequently, the results are not fully generalizable and have limitations in their external validity.

The building of four groups based on the elapsed time since surgery was a decision taken within the PFF expert group. This was a pragmatic and practical decision, but it has to be acknowledged that other parameters (e.g., ADLs, function) may qualify better for the definition of recovery groups than time since surgery.

The current paper focuses on walking amount, pace, and pattern. The output of the full DMO matrix of Mobilise-D includes results on rhythm and bout-to-bout variability. As the investigation of the clinical usefulness of rhythm and bout-to-bout variability is ongoing and currently considered exploratory, these outcomes were not included.

Most of the walking bouts during the acute phase occurred during physiotherapy when participants had assistance or were accompanied by nursing staff. Hence, further technical validation of spatial parameters in acute PFF patients is needed to test their validity in this phase. This has not been done in the Mobilise-D technical validation study due to the COVID-19 pandemic. Unfortunately, it was not possible to establish a procedure to distinguish independent from supervised walking bouts, simply because of limited personnel availability. Lastly, useful information on individuals’ living environment would have been an important addition to the protocol due to the considerable influence it has on daily mobility and routines. However, this was not implemented again, due to lack of personnel to visit study participants’ homes that were sometimes far away from the clinics.

## Perspectives

Several publications are in preparation. Upcoming papers will address construct validity of DMOs by describing the relationship with clinically relevant constructs such as PROMs and COAs. The assessment of the ability of DMOs and physical capacity measures to detect change and the estimation of minimal important differences (MID) of DMOs to measure change in disease (worsening or improvement) is also in the process to be submitted. Further work will cover the predictive capacity of DMOs on care home admission and mortality. We also plan to publish the longitudinal data as soon as possible. In these analyses, we will also consider multivariate modelling to identify predictors of recovery or validate DMOs’ sensitivity to clinical status. Qualitative data will be published on subjective experiences of patient journeys. Environmental data such as meteorological data will be analysed to understand their impact on mobility. Further ongoing work will include the examination of mobility trajectories of patients with different types of implants, since these can be expected to affect outcomes of recovery. We strongly encourage similar work on PFF patients with dementia and delirium living at home and in long-term care. Another knowledge gap is comparative effectiveness research across service models and countries.

## Conclusions

The observed difference in walking amount, pace, and pattern across recovery phases indicate that DMOs can provide an in-depth analysis of real-world mobility of hip fracture survivors. When confirmed by longitudinal analyses, including results on minimal important differences, the use of selected DMOs will provide a novel approach for monitoring, predictive modeling, prognosis, stratification, and evaluation of clinical trials and hip fracture services.

## Data Availability

The data used in the preparation of this paper will be available to download from the Mobilise-D Zenodo page from late 2025 (https://zenodo.org/communities/mobilise-d).
